# Efficacy and intrarenal pressure analysis of flexible and navigable suction ureteral access sheaths with flexible ureteroscopy in modified surgical positions for 2–6 cm upper urinary tract stones: a multicenter retrospective study

**DOI:** 10.3389/fmed.2024.1501464

**Published:** 2024-11-20

**Authors:** Junjie Bai, Tong Shangguan, Gaoyu Zou, Liangguang Liu, Xiyun Xue, Jun Lin, Yushi Ye, Xiuwu Ruan, Yongbin Li, Shengzeng Yang, Yangjian Chi, Yongqiang Nian, Xingxiang Chen, Rong Liu, Weizhong Cai, Shaoxing Zhu, Jianhui Chen

**Affiliations:** ^1^Department of Urology, Fujian Medical University Union Hospital, Fuzhou, China; ^2^Graduate of School, Fujian Medical University, Fuzhou, China; ^3^Department of Urology, Luoyuan County Hospital, Fuzhou, China; ^4^Department of Urology, Fuzhou Taijiang District Hospital, Fuzhou, China; ^5^Department of Urology, Jian’ou Municipal Hospital, Nanping, China; ^6^Department of Urology, Fuqing Second Hospital, Fuzhou, China; ^7^Department of Urology, Fujian Medical University Union Hospital Pingtan Branch, Fuzhou, China

**Keywords:** retrograde intrarenal surgery, flexible and navigable suction ureteral access sheaths, upper urinary tract stones, intrarenal pressure, reverse trendelenburg lithotomy position

## Abstract

This multicenter retrospective study aimed to assess the efficacy, intrarenal pressure (IRP), and complications of retrograde intrarenal surgery (RIRS) using a flexible and navigable suction ureteral access sheaths (FANS-UAS) in the reverse Trendelenburg lithotomy position (RTLP) for treating kidney and upper ureteral stones measuring 2–6 cm. Conducted at six medical centers in Fujian Province from 2022 to 2024, the study included 231 patients with a median stone size of 26 mm. The immediate stone-free rate (ISFR) was 90.48%, while the SFR at postoperative day 30 was 95.67%. Only two patients developed postoperative fever, two patients had ureteral laceration and most experienced mild pain. Although surgical time increased with stone size, factors such as sex, infundibulopelvic angle (IPA), and stone density had little effect on duration, and there was no significant difference between ISFR and 30-day SFR. Importantly, all IRP measurements remained within normal limits. These findings suggest that RIRS with FANS-UAS in RTLP is a safe and effective approach for managing upper urinary tract stones of 2–6 cm, especially in 2–4 cm stones.

## Introduction

Urolithiasis is a common urological disease, with global incidence rates of 5 to 20%, and a prevalence of 6.4% in China, peaking at 11.6% in the southern regions (1–4). Its high recurrence rate of up to 50% and negative effects on kidney function make it a major public health issue (5–8).

With advancements in diagnostic and therapeutic technologies, traditional open surgery for upper urinary tract stones has gradually been replaced by minimally invasive approaches. The European Association of Urology (EAU) recommends Retrograde Intrarenal Surgery (RIRS) as the preferred treatment for kidney stones <2 cm, owing to its minimally invasive nature, low risk, and fast recovery (9). Percutaneous nephrolithotomy (PCNL) is an invasive procedure that may lead to serious complications such as renal bleeding and damage to adjacent organs (10), if PCNL is contraindicated, RIRS can also be an alternative (9). With advancements in laser lithotripsy technology and negative pressure suction equipment, the effectiveness and safety of RIRS for managing complex upper urinary tract stones have been confirmed. In patients with stones larger than 60 mm, the long-term stone-free rate (LSFR) after multiple RIRS sessions can reach 48% (11–14). However, the main limitations of this surgery include the limited stone-free rate (SFR), the need for multiple procedures, and life-threatening complications associated with intrarenal pressure, such as urosepsis (15).

The ureteral access sheath (UAS) was developed for RIRS procedures to facilitate movement of the flexible ureteroscope (FURS), reduce surgical time, lower intrarenal pressure (IRP), and improve the stone-free rate (SFR) (16). Traditional UAS (TUAS) is non-flexible, and its tip placement is not standardized in guidelines, which is often positioned in the upper ureter rather than the renal pelvis or calices. The expert consensus suggests that we should place the tip of the TUAS 2 cm below the ureteropelvic junction (UPJ) (17). However, the gap between the tip of TUAS and FURS can become obstructed by ureteral mucosa or stone fragments, which may interfere with the outflow of irrigation fluid from the upper ureter and ultimately lead to increased IRP (18, 19). A new type of flexible and navigable suction ureteral access sheaths (FANS-UAS) has been successfully developed and applied clinically, with a 10 cm flexible tip area that can be adjusted to enter the target calyx as needed. On the basis of maintaining a low IRP, reducing the number of bacteria and absorbing endotoxin substrates, the adjustable continuous negative pressure suction ensures sufficient irrigation speed and maintains a clear surgical field of view, while effectively removing fragmented stones and dust, and reducing the thermal energy generated by laser lithotripsy (20).

Studies have shown that using a FANS-UAS for treating upper urinary tract stones ≤4 cm, while extending surgery time, achieves better immediate SFR (ISFR), LSFR and safety compared to TUAS (21). However, there is a lack of research data on the impact of FANS-UAS on IRP, extended surgery time, and methods to improve SFR. Therefore, it is necessary to explore and improve this technique to enhance its safety and surgical efficiency. Based on previous animal studies, we found that the reverse trendelenburg position effectively reduces IRP and thus lowers the incidence of complications (22). Building on this, the study combines the reverse trendelenburg lithotomy position (RTLP) with FANS-UAS for RIRS in treatment of 2–6 cm upper urinary tract stones, and analyzes the effectiveness, safety, and intraoperative IRP.

## Materials and methods

### Patients

**Figure 1:**
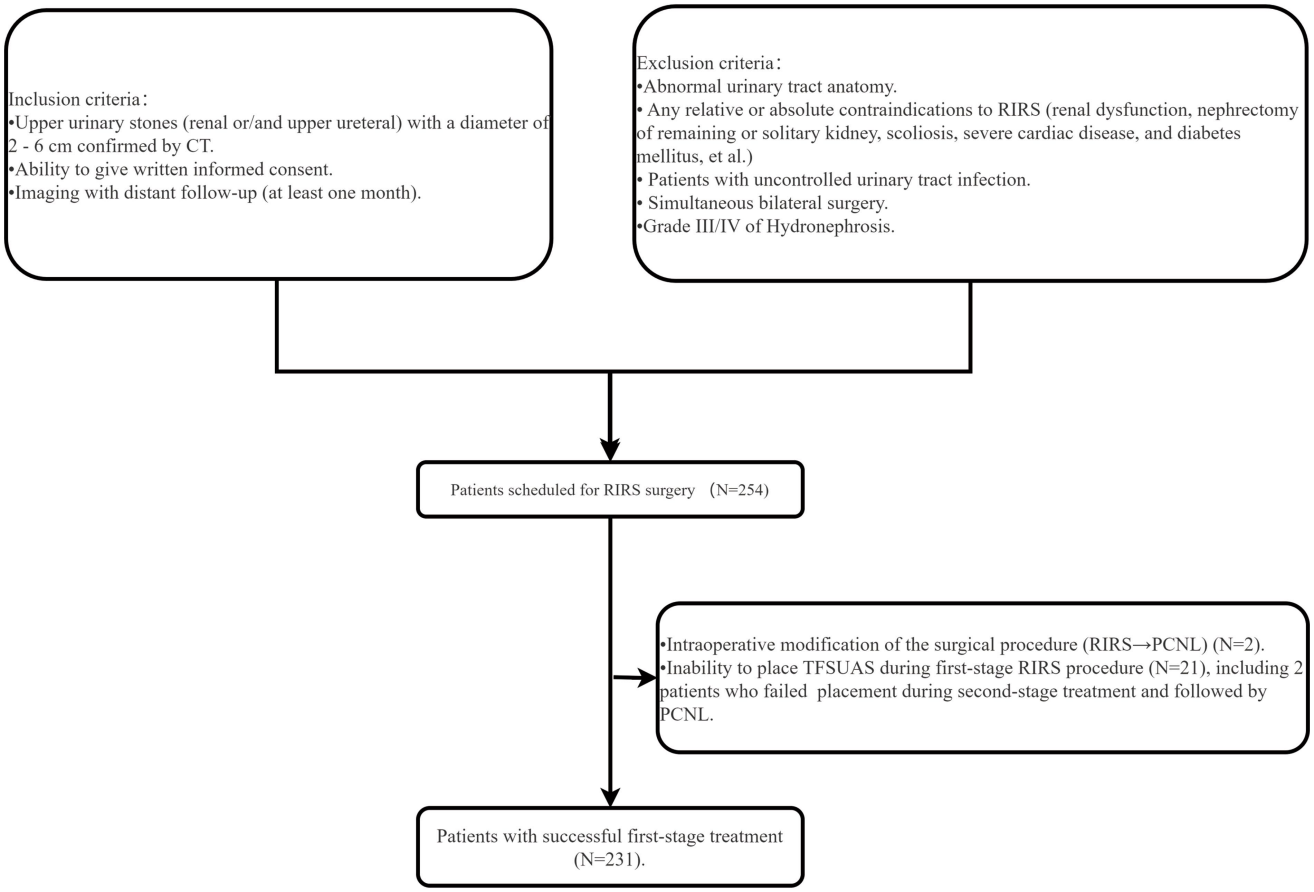
Patient screening flowchart.

In this study, we retrospectively analyzed data from patients with upper urinary tract stones treated at six medical centers in the Fujian region, including five primary healthcare facilities, between May 2022 and May 2024. Based on the inclusion and exclusion criteria, a total of 254 patients were scheduled for RIRS. Of these, two patients had their surgical approach converted from RIRS to PCNL during the first-stage procedure. Additionally, 21 patients experienced failed FANS-UAS placement during initial surgery, leading to postponement of their procedures, and two patients underwent PCNL after failed FANS-UAS placement during the second-stage surgery (Figure 1). Ultimately, a total of 231 patients were included in the study, including 80 patients from primary healthcare centers [Fujian Medical University Union Hospital (*n* = 151), Luoyuan County General Hospital (*n* = 14), Fuqing City Second Hospital (*n* = 14), Jian’ou City Municipal Hospital (*n* = 21), Fujian Medical University Union Hospital Pingtan Branch (*n* = 18), and Taijiang Hospital of Fuzhou City (*n* = 13)]. Clinical data and surgical procedures were collected for analysis during the perioperative period. All patients underwent urinary non-contrast computed tomography (CT), with additional IVU or CTU exams performed to accurately assess the ureter, renal pelvis, and infundibulopelvic angle (IPA). The stone burden and Hounsfield Density was assessed using CT in bone window mode. The degree of hydronephrosis was graded according to the SFU grading system (23). According to the “Chinese Guidelines for Diagnosis and Treatment of Urological and Andrological Diseases,” preoperative antibiotics were given based on urine analysis and midstream urine culture results. Patients with positive cultures received antibiotics per sensitivity tests until negative results were achieved. Those with negative cultures but positive leukocyte and/or nitrite tests received antibiotics based on local susceptibility for at least 3 days. Patients with negative cultures received a single dose of prophylactic antibiotics 1 h before surgery. Stone size was measured in its largest diameter. When multiple stones presented, the sum of diameters was recorded as stone burden. The study was approved by the ethics committees of all centers for case data collection and the Declaration of Helsinki was strictly adhered to during the study.

### Surgical techniques

**Figure 2:**
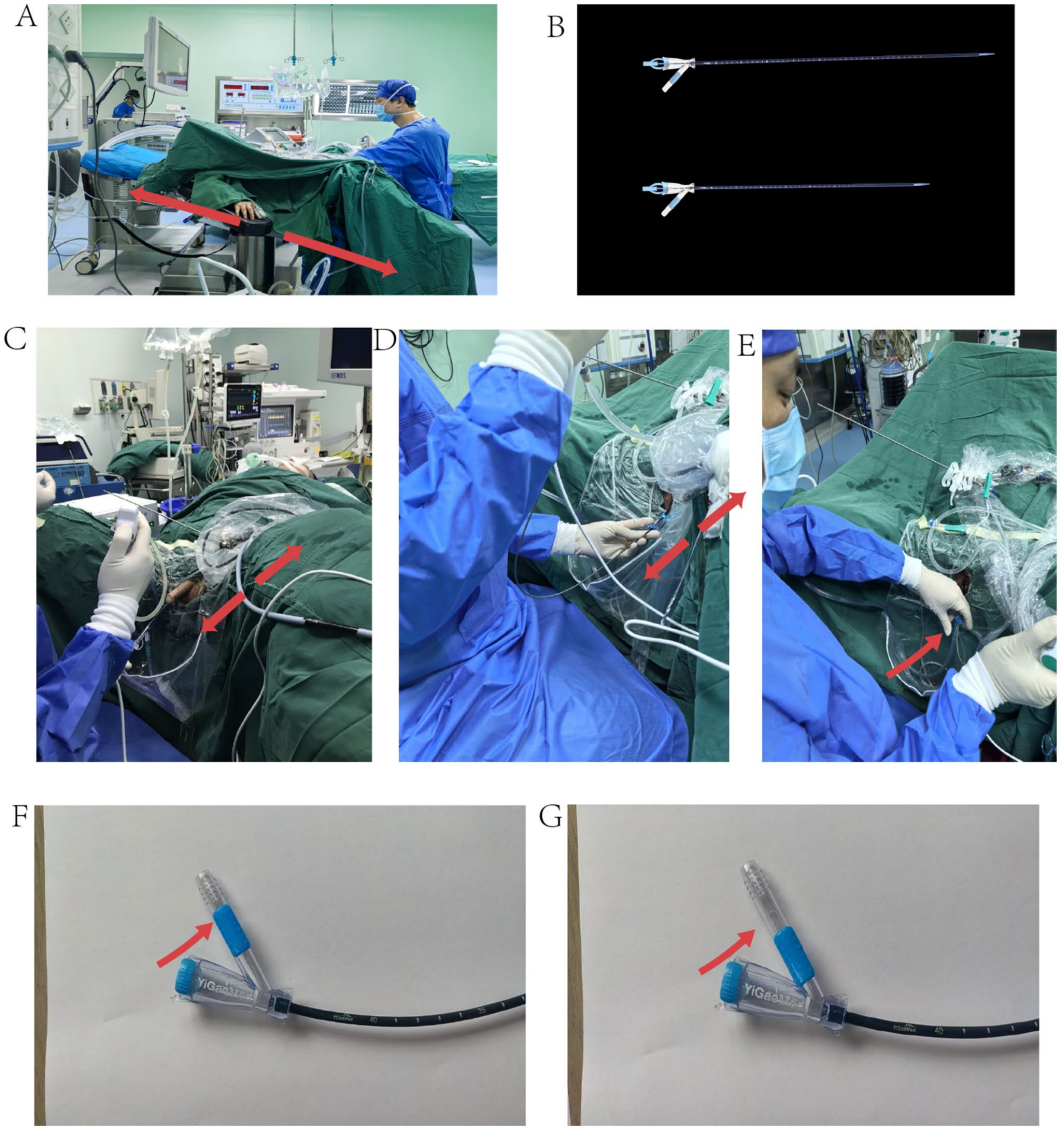
Surgical position and FANS-UASA. (A) Reverse Trendelenburg lithotomy position (20°). (B) Male FANS-UAS (Above), Female FANS-UAS (Below). (C) FANS-UAS penile portion and extracorporeal portion in the same plane as the operating table. (D) The extracorporeal portion of the FANS-UAS is in the same plane as the operating table. (E) Left hand manages FURS movement and adjusts suction. (F) FANS-UAS fully closed for suction. (G) FANS-UAS fully open, no suction.

**Figure 3:**
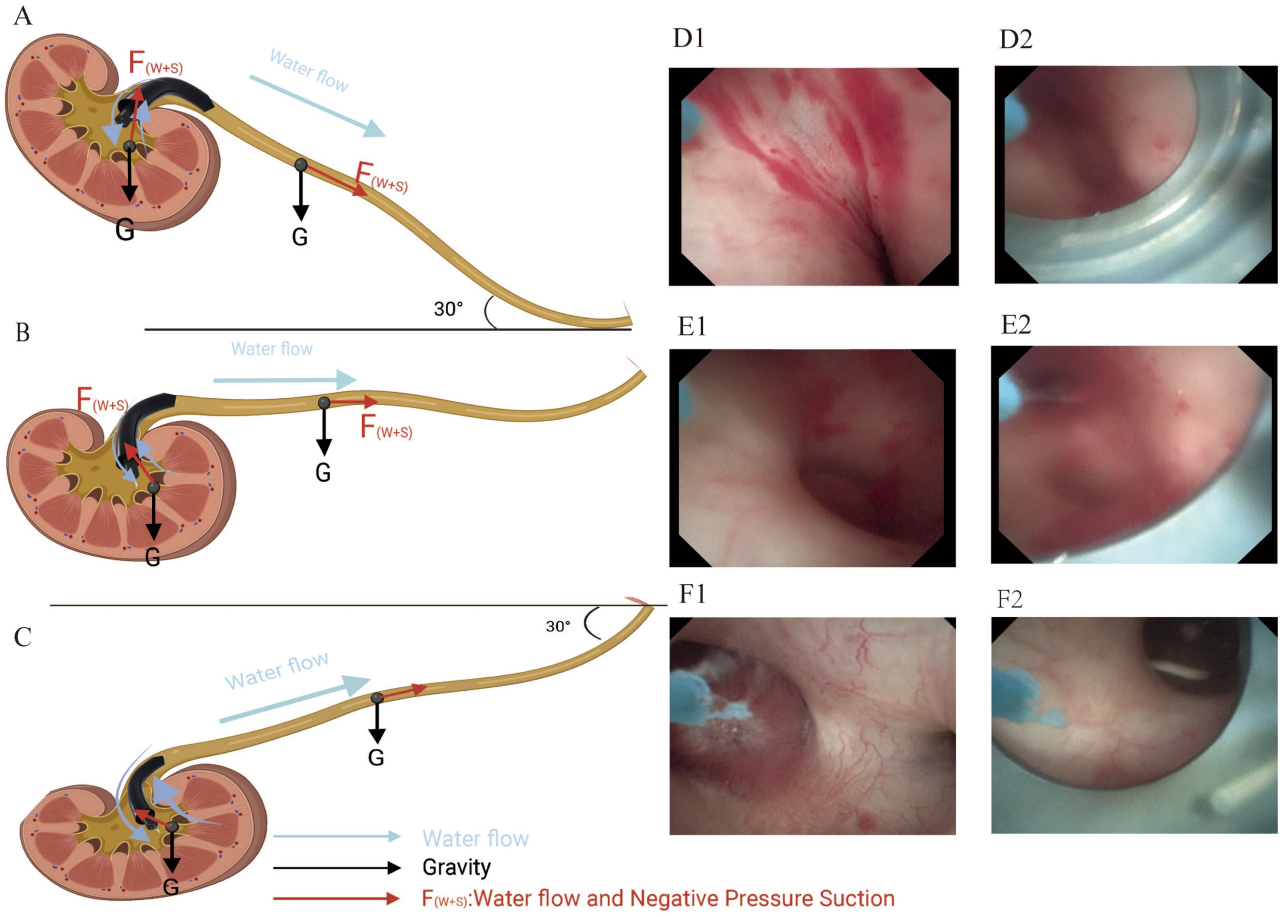
Mechanical analysis of renal and ureteral stones in different surgical positions and the degree of dilatation of the renal pelvis and calyces in the sagittal plane. (A) RTLP (30°). (B) Spine position. (C) TLP (30°). The length of the arrows in red and black in the figure represent the magnitude of the force on the stone. (D1/D2) Renal pelvis and calyces are in a collapsed state. (E1/E2) renal pelvis, and calyces are in a mildly collapsed state. (F1//F2) renal pelvis and calyces are in a dilated state.

All procedures are performed by designated experienced urologists (experience in at least 100 surgeries). After satisfactory general anesthesia, the RTLP (30°) was applied for all patients (Figures 2A, 3). A semi-rigid ureteroscope is inserted into the upper ureter or renal pelvis with the aid of a 0.028-inch ultra-smooth guidewire. The ureteroscope is then removed, leaving the guidewire in place. A 12/14 Fr FANS-UAS (Elephant II, Zhejiang YiGao Medical Technology Co. Ltd., Hangzhou, China) is inserted, with a 50 cm sheath for males and a 40 cm sheath for females (Figure 2B). The digital FURS (Uscope 9.2 Fr, PUSEN, Zhuhai, China) is then inserted through the FANS-UAS, and the end of the FANS-UAS is adjusted at the ureteropelvic junction (UPJ) under direct vision until the tips of both the FURS and FANS-UAS are positioned in the renal pelvis or calyx near the stone. During lithotripsy, ensure that the cephalic end of the FURS is level with or slightly medial to the cephalic end of the FANS-UAS. The extracorporeal portion of the TUSUAS is maintained at the same level as the operating table (Figures 2C,D). The irrigation flow rate of the flushing pressure pump was set to 100 ml/min. The FANS-UAS was connected to the vacuum device, with the negative pressure set to 0.02 MPa. A 272 μm holmium laser fiber (Raykeen laser Technology Limited Corporation, Shanghai, China) was used to crush stones with a setting of 1.0 to 2.0 J energy and a frequency of 15 to 25 Hz. The pressure-regulated vent is maintained at maximum opening, and the urologist adjusts the actual intraoperative negative pressure through the pressure-regulated vent on a case-by-case basis in order to maintain the pelvic mucosa in the field of view in a state that is amenable to surgical manipulation and mildly collapsed (Figures 2E–G). During lithotripsy, the FURS was repeatedly advanced and withdrawn to remove stone fragments via the flushing fluid. After clearing the renal pelvis and calyces of stones, the integrity of the renal pelvic mucosa was confirmed, with no significant bleeding. The degree of ureteral injury was determined according to the Traxer grading system (24): grade 0 was no damage or only bleeding spots on the mucosa of the inner wall of the ureter; grade 1 was slight damage to the mucosa of the inner wall of the ureter without involvement of the muscle layer; grade 2 was a tear of the mucosa and smooth muscle of the inner wall of the ureter, with an intact outer membrane (periureteral fat was not seen); grade 3 was a Tear of the mucosa and smooth muscle of the inner wall of the ureter with perforation of the outer membrane (periureteral fat is seen); grade 4 is complete avulsion of the ureter.

A guidewire was left in place, the scope was removed, and a double-J stent was inserted along the guidewire, correctly positioned under endoscopic vision. The operation time was measured from the insertion of FURS into the urethra until its removal.

A CNC system based on sheath-side fiber optic pressure sensor monitoring is used for IRP monitoring, where the fiber optic pressure sensor enters the renal pelvis through a side channel to monitor renal pelvis pressure (CN109998698A/CN201910273371.X). IRP measurements were performed on 30 patients who consented to treatment at the Union Hospital of Fujian Medical University. A 200-μm fiber optic was used to measure IRP every 30 min at the upper, middle, and lower calyces, as well as the upper ureter. Measurements were recorded with the vent fully open or closed, and the highest peak pressure, fluctuating between 1 and 3 mmHg, was noted.

### Postoperative management

Based on preoperative findings, routine postoperative treatment included anti-infective therapy and rehydration with routine blood tests, C-reactive protein (CRP), procalcitonin (PCT) and biochemical evaluation of renal function. On the first postoperative day, the location of the double J-tube and stone clearance rate (SFR) were assessed by abdominal plain radiographs (kidney–ureter-bladder, KUB) and urologic CT. Renal stones <2 mm in diameter were defined as clinically insignificant and considered cleared (25). CT review was performed 1 month postoperatively, and the double J-tube was removed if the stone was completely cleared, or secondary lithotripsy was performed if there was a residual stone. The SFR evaluated endoscopically at the end of the procedure and through imaging on the first postoperative day is referred to as the immediate SFR (ISFR), while the SFR assessed via imaging 30 days or later is referred to as the long-term SFR (LSFR).

The duration of the procedure was calculated from the start of lithotripsy to the end of the procedure. Assess for complications during and after surgery, and patient response within 24 h after surgery was assessed using the numerical rating scale for pain (NRS Pain), with 0 indicating none and 10 indicating the most severe. 1–4 was classified as mild, 5–8 as moderate, and 9–10 as severe. Thirty patients treated at the Union Hospital of Fujian Medical University agreed to undergo intraoperative intrarenal pressure monitoring.

### Statistical analysis

All statistical analyses were performed using SPSS 26.0. Descriptive analysis was used to assess the distribution patterns of patient demographics, stone characteristics, and surgical data. Categorical variables were expressed as percentages. The chi-square test or Fisher’s exact test was used to compare categorical variables. A difference was considered statistically significant at *p* < 0.05.

## Results

Demographics and preoperative data are shown in Table 1. Overall, out of 231 patients, there were 147 males and 84 females with a median age of 55 (range: 21–80) years. The median BMI was 23.52 (range:17.95–31.88) kg/m^2^. All patients had indications for surgery, with no absolute contraindications, and all had a stone burden of ≥2.0 cm. There were 153 patients with renal calculi only and 29 cases with ureteral calculi only. The other 49 cases were diagnosed with concomitant renal and ureteral calculi. And 127 patients had stones in the lower calyces. Preoperatively, 62 cases had no hydronephrosis and 161 patients diagnosed with varying degrees of pyelonephrosis. The median stone size was 26 (range: 20–57) mm with a median stone density of 1,109.00 (range: 619–2,204) Hounsfield units (Hu). The meidan IPA was 44.00° (range: 6.4°–100.9°). There were 115 cases with negative white blood cell count in preoperative urine routine, 60 cases with (+), 16 cases with (++), 19 cases with (+++). Seventeen patients had positive urine cultures and received antibiotics until the urine cultures were negative before proceeding to surgery, 17 of these patients had positive urine cultures.

**Table 1:** Peri-operative data.

Variables	Values
Age(years)	55.00 [43.50, 64.00]
Gender, *n* (%)	
Male	147 (63.64%)
Female	84 (36.36%)
BMI (kg/m^2^)	23.52 [22.01, 25.27]
Stone size (mm)	2.60 [2.30, 3.30]
Side, *n* (%)	
Left	132 (57.14%)
Right	99 (42.86%)
Grade of Hydronephrosis, *n* (%)^a^	
Grade 0	62 (26.84%)
Grade I	70 (30.30%)
Grade II	99 (42.86%)
Positive urine culture, *n* (%)	17 (7.36%)
Preoperative URI, *n* (%)	
Negative	128 (55.41%)
1+	60 (25.97%)
2+	22 (9.52%)
3+	21 (9.09%)
Hounsfield density (Hu)	1,109.00 [1,000.00, 1,241.00]
Stone position, *n* (%)	
Upper calyx	5 (2.16%)
Middle calyx	22 (9.52%)
Lower calyx	38 (16.45%)
Upper ureter	29 (12.55%)
Renal and ureteral stone	49 (21.21%)
Multiple calyxes	88 (38.10%)
Contain Upper calyx, *n* (%)	127 (54.98%)
IPA (°)	44.00 [31.40, 55.30]

BMI, body mass index; UTI, urinary tract infection; IPA, infundibulopelvic angle; Data are presented as median (first quartile, third quartile), or number (proportion).

a SFU grading system.

Table 2 summarizes the postoperative treatment outcomes and complications of the patients, with mean operative time was 126.81 ± 57.01 min. ISFR was achieved in 90.48% (209/231) cases and 30-days SFR was 95.67% (221/231). The overall complication rate was extremely lower (1.72%, 4/231). Post-operative fever occurred in 2 patients (0.86%) with 5.5 cm infectious stones and 4.4 cm infectious stones and it was successfully managed by potent antibiotics. Two patients (0.86%) had grade II ureteral wall injuries during surgery, which compromised first-time outcomes. Medical staff performed NRS pain scoring on postoperative patients within 24 h, 95.24% (220/231) patients had mild pain. Eight patients with residual stones were re-treated with RIRS at 1 month and completely cleared of stones, and 2 patients did not undergo second-stage treatment, resulting in a final 90-day SFR of 99.57%.

**Table 2:** Clinical outcomes postoperatively in 231 patients.

Variables	Values
Immediate SFR, *n* (%)	209 (90.48%)
SFR at postoperative day 30, *n* (%)	221 (95.67%)
Operation time(min)	125.98 ± 56.87
Postoperative hospitalization (days)	1.13 ± 0.41
Total complication, *n* (%)	
Fever	2 (0.86%)
Septic shock	0
Intraoperative bleeding	0
Transient hematuria	0
Ureteral wall injuries (Grade I)	0
Ureteral wall injuries (Grade II)	2 (0.86%)
Ureteral wall injuries (Grade III/IV)	0
NRS pain	
1	30 (12.99%)
2	86 (37.23%)
3	103 (44.59%)
4	1 (0.43%)
5	11 (4.76%)
Residual stone treatment	
None	2 (20%)
ESWL	0 (0%)
RIRS	8 (80%)
SFR at postoperative day 90, n (%)	229 (99.57%)

**Figure 4:**
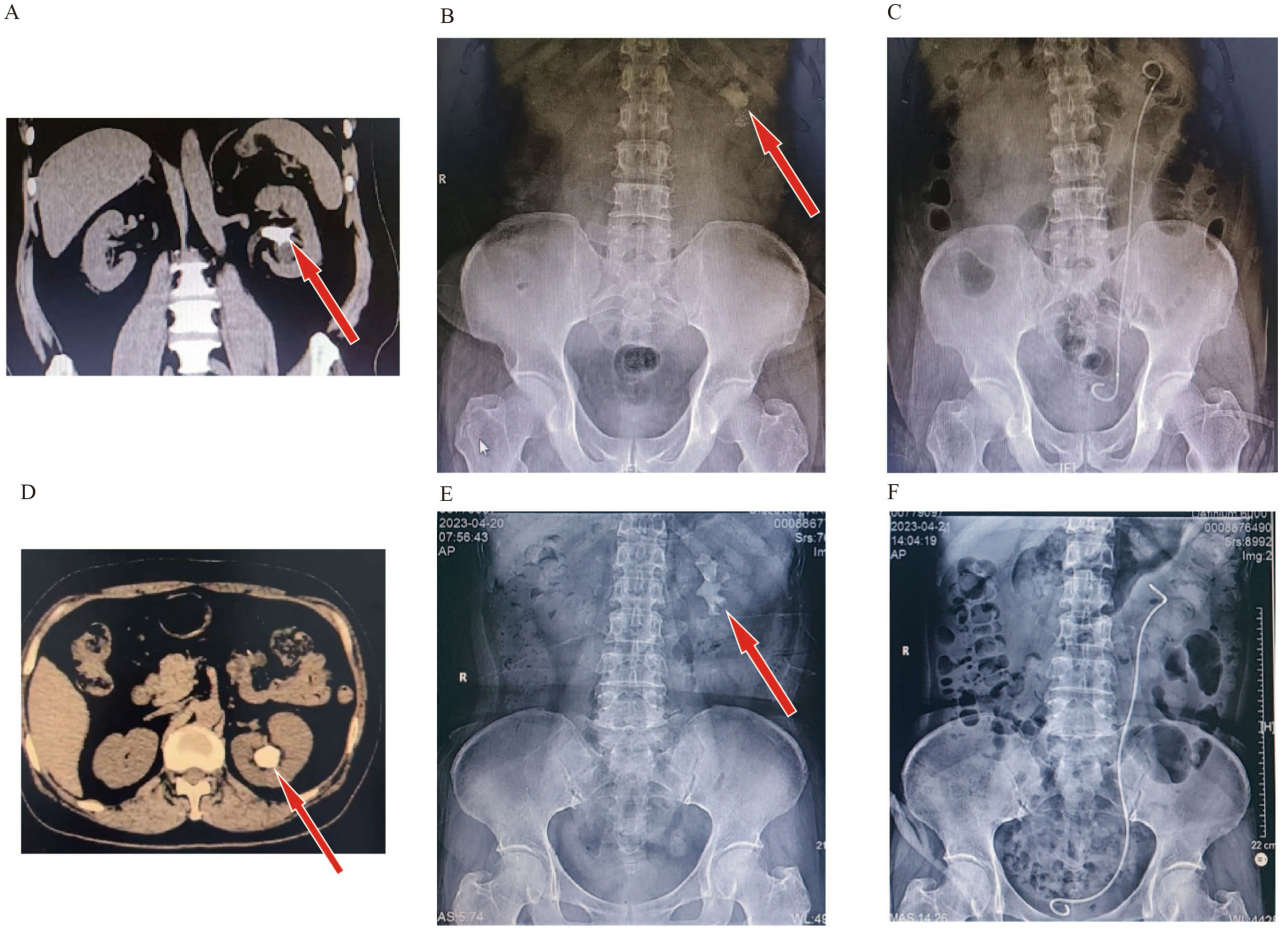
Comparison of preoperative and postoperative imaging in one-stage surgery for large stones. (A,B) Patient with 5.0 cm kidney stone (Preoperation). (C) Patient with 5.0 cm kidney stone (Postoperation). (D,E) Patient with 5.6 cm kidney stone (Preoperation). (F) Patient with 5.6 cm kidney stone (Postoperation).

Table 3 analyzes the results of stratification according to stone size. Surgical time increased with increasing stone size, and ISFR and 30-days SFR decreased with increasing stone size. Patients with stones 4–6 cm in size still had up to 80.6% ISFR and 30-day SFR (Figure 4). Gender, stone density and IPA were analyzed on this basis (Table 4), and the results showed that there was no significant difference between genders in terms of operative time and SFR. When stone size was 5–6 cm, IPA showed a statistically significant difference in operative time but not in SFR. When stone size was 2–3 cm and 5–6 cm, there was a significant effect of stone density on operative time (*p* < 0.05).

**Table 3:** Perioperative data stratified according to stone size.

Variables	*n*	Operation time(min)	Immediate SFR	SFR 30 days postoperative
Stone size				
2 ~ 3 cm	150	92.23 ± 16.78	94.0%	100%
3 ~ 4 cm	50	154.38 ± 19.65	86.0%	92.0%
4 ~ 5 cm	24	226.33 ± 18.01	83.3%	83.3%
5 ~ 6 cm	7	302.43 ± 28.06^*^	71.4%^*^	71.4%^*^

^*^p < 0.05.

**Table 4:** Perioperative data stratified according to stone size.

	2–3 cm	ISFR	30-day SFR	3–4 cm	ISFR	30-day SFR	4–5 cm	ISFR	30-day SFR	5–6 cm	ISFR	30-day SFR
Gender		94.0	100		86.0	92.0		83.3	83.3		71.4	71.4
Male	93.60 ± 17.17	95.2	100	158.05 ± 19.02	81.8	95.5	230.07 ± 17.55	86.7	86.7	306.80 ± 32.71	80.0	80.0
Female	89.02 ± 15.55	91.1	100	151.50 ± 20.00	89.3	89.3	220.11 ± 18.00	77.8	77.8	291.50 ± 10.61	50.0	50.0
IPA		94.0	100		86.0	92.0		83.3	83.3		71.4	71.4
<30°	90.33 ± 16.23	97.0	100	160.00 ± 16.83	90.0	90.0	224.50 ± 15.20	75.0	75.0	315.00 ± 31.27	50	50
>30°	92.76 ± 16.97	93.2	100	152.97 ± 20.24	85.0	92.5	226.70 ± 18.85	85.0	85.0	285.67 ± 12.58B	100	100
IPA		94.0	100		86.0	92.0		83.3	83.3		71.4	71.4
<45°	90.23 ± 17.20	94.0	100	153.39 ± 16.80	91.3	91.3	229.85 ± 18.66	84.6	84.6	315.00 ± 31.27	50	50
>45°	94.77 ± 16.00	93.9	100	155.22 ± 22.08	81.5	92.6	222.18 ± 17.13	81.8	81.8	285.67 ± 12.58B	100	100
Density		94.0	100		86.0	92.0		83.3	83.3		71.4	71.4
<1,100	88.87 ± 17.59	94.7	100	153.70 ± 19.07	95.0	95.0	220.64 ± 17.06	85.7	85.7	279.00 ± 7.07	100	100
>1,100	95.68 ± 15.27C	93.2	100	154.83 ± 20.34	80.0	90.0	234.30 ± 16.96	80.0	80.0	311.80 ± 28.01C	60.0	60.0

A: comparison with male, *p* < 0.05; B: comparison with <30°, *p* < 0.05; C: comparison with < 1,100 Hu, *p* < 0.05.

**Figure 5:**
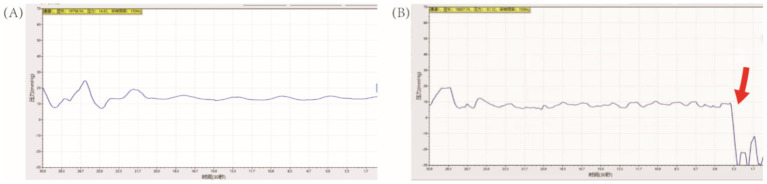
Monitoring of intrarenal pressure during RIRS. (A) Irrigation without suction. (B) Irrigation with fully suction, indicated by the red arrow.

Table 5 summarizes the results of IRP monitoring in 30 patients during surgery. During the lithotripsy process, pressures varied across the upper calyx, middle calyx, lower calyx, and upper ureter, with statistically significant differences. The IRP at each location during the perfusion period, respectively, was 19.82 ± 0.57 mmHg, 18.07 ± 0.85 mmHg, 20.32 ± 0.72 mmHg, and 21.59 ± 1.14 mmHg (*p* < 0.05). After negative pressure suction is initiated, the pressure drops rapidly and falls below -30 mmHg (Figure 5).

**Table 5:** Intrarenal pressures at different renal calyces and upper ureter during RIRS.

Position	Upper	Middle	Lower	Upper ureter	*p* value
IRP (mmHg), Mean ± SD	19.82 ± 0.57	18.07 ± 0.85a	20.32 ± 0.72b	21.59 ± 1.14abc	<0.001*

a: *p* value < 0.05 compared with upper; b: *p* value < 0.05 compared with middle; c: *p* value < 0.05 compared with lower.

## Discussion

For kidney and ureteral stones with a diameter >2 cm, PCNL is recommended as the preferred treatment method by the American Association of Urology (AUA) and the EAU guidelines (9, 26), but it carries high risks and a long learning curve (10, 27). With recent technological advancements in FURS, such as the introduction of digital ureteroscopes, UAS, and laser technology, there has been a reduction in surgical complications for patients while maintaining good SFR. These advancements have expanded the indications for RIRS, allowing it to be used for the treatment of large and complex stones (28, 29). When using TUAS, clinicians have encountered certain limitations. Firstly, it cannot immediately clear fragments, and up to 38% of renal units may still have residual fragments >2 mm after CT scan evaluation, which increases the likelihood of repeat surgery by 9 times (30–33), The ability to extract small renal stones can reduce the risk of subsequent emergency department visits, surgeries, and stone regrowth (34). Secondly, stone fragments may obstruct the clear surgical field and the UAS outlet (35), necessitating increased irrigation flow. Increased irrigation and small stone blockage can lead to elevated IRP, and prolonged surgery and elevated IRP can usually cause pyelovenous reflux, ultimately increasing the risk of sepsis (36). A novel FUAS has the potential to expand the indications for FURS by addressing issues such as residual stone fragments, high IRP, low efficiency, and insufficient surgical vision. This technology offers a promising solution to these challenges.

Compared to TUAS, the novel FUAS has greatly improved efficiency. When treating stones ≤3 cm, the SFR for the FANS-UAS group was higher immediately after surgery and at 3 months (81.3 vs. 49.4% and 87.5 vs. 70.0%), with fewer postoperative complications and a significant improvement in quality of life. When treating stones between 2 and 4 cm, ISFR and LSFR reached higher rates of 87.20 and 95.20%, with an overall complication rate of only 1.6% (37, 38). In our study, the overall ISFR was 90.48%, 30-days SFR was 95.67% and the overall complication rate was 1.72%, the lower complication may be due to the gravitational effect of the RTLP position and the maintenance of the extracorporeal position of the FANS-UAS, where the ureter is straightened during the procedure thereby facilitating the access of the FANS-UAS and the drainage of the lithotripsy and the instillation fluid. However, SFRs greater than 4 cm were still limited (80.6%), which may be a stone size to consider for RIRS selection.

The enhancement of SFR and reduction of complications in RIRS are mainly due to the advantages of FUAS. Firstly, FANS-UAS has a wide range of bending angles, allowing access to the renal pelvis, middle upper calyx, and most lower calyces while retaining the flexibility and maneuverability of the ureteroscope. Statistics show that about 35% of renal stones are located in the lower calyx, which is difficult to treat due to the complexity of its anatomical structure, prolonging surgery time and limiting SFR (39). In renal anatomical parameters, the IPA has been reported to affect treatment success, with an inclination of <45 degrees often indicating treatment failure, let alone 30 degree (40–42). Therefore, FANS-UAS can solve this problem to some extent. In our study, the effect of IPA <30° and IPA <45° was only significant in terms of operative time for 5–6 cm stones. Smaller IPA still showed lower SFR, although there was no statistical difference. Still this also suggests that both FUAS and disposable electronic ureteral flexible scopes are limited in length and maximum curvature, and that it may still not be possible to reach the lower calyces when the IPA is too small, in which case, therefore, the use of stone-sleeving baskets is increased (43). A sheath basket was not used in our study, which placed the sheath opening as close as possible to the neck of the renal calyx in order to address these stones when the IPA was too sharp for direct access. A ureteroscope was inserted into the renal calyx with a moderate increase in irrigation flow and high-pressure irrigation was used to help flush out the stones. Second, the FANS-UAS is able to utilize negative pressure suction. It has been reported that the IRP tends to exceed 30 mmHg with the 12/14 Fr TUAS, which can affect the visual field if the irrigation flow is controlled (22, 44), Ostergar et al. verified that UAS with negative pressure suction was effective in reducing intrarenal pelvic pressure (45), Peng et al. preliminarily validated the efficacy and safety of FANS-UAS in children, but as of now, there is still no FANS-UAS in adult patients and patients with high stone loads. In our study, IRP was recorded and analyzed using a fiber-optic pressure transducer-based intra-pelvic pressure monitoring device, and the maximal IRP at individual renal calyces and UPJs was below the threshold during the procedure, and the pressure dropped below −30 mmHg during negative pressure aspiration. However, we believe that the IRP in individual renal calyces does not represent the IRP of the entire renal collecting system, because when the FURS and FUAS are at the same level, the IRP in the calyces is also slightly elevated as a result due to the high flow of perfusate at the head end of the FUAS (46), it is still safe. Based on this assurance that IRP is in the normal range, adjusting the negative pressure value and perfusion rate to increase vortex formation, adjusting the surgical position, and thereby elevating the SFR can be attempted. In addition to this, effective irrigation fluid circulation and negative pressure suction effectively removes stone fragments, maintains a clear field of vision, and prevents laser energy-induced damage to the mucous membranes of the renal pelvis and calyces. It has been shown that as the degree of hydronephrosis increases to moderately severe, the lower the ureteroscopic lithotripsy stage I stone clearance rate is, with an overall clearance rate of approximately 61.5–69.7% (47). Similarly, postoperative hydronephrosis has been associated with difficulty in stone evacuation, and studies have shown that preoperative coexisting hydronephrosis is an independent predictor of difficulty in spontaneous removal of residual renal stone fragments after ureteral flexible ureteroscopy, and that patients with moderately severe hydronephrosis are more likely to have a complication of ureteral stricture in the postoperative period, which can lead to difficulty in stone evacuation (48, 49). Our study excluded patients with Grade III/IV hydronephrosis, so our ISFR and LSFR in stage 1 were higher than in previous studies, and many of the stones that could not be aspirated during the procedure because of the small IPA and being in the lower calyces of the renal pelvis were successfully expelled from the body postoperatively.

In our study, we analyzed the factors influencing the duration of surgery, ISFR and LSFR. We found that men would have a longer operation time than women, although there was no statistical difference. This may be related to the physiology of the male urethra, where 3 urethral strictures and 2 urethral curvatures allowed additional procedure time to be consumed while withdrawing the scope frequently to remove stones. Therefore, during the procedure, we maintained the penis in the same plane as the body to ensure patency of the FUAS and minimize unnecessary time loss. Hounsfield density used to be an important parameter in predicting the outcome of RIRS (50). Oztekin et al. concluded that stone density > 1,100 HU was an independent predictor of RIRS failure (51). In our study, the Hounsfield density was significantly different only for 2–3 and 5–6 cm stones in terms of operative time and not in terms of SFR, which may be related to the laser settings, which can be adjusted by the surgeon according to the actual situation during the operation, the use of higher frequencies (>25 Hz) may also be considered.diyi Compared to other studies (37, 43). Our longer operative times are related to the fact that we spend more time suctioning for lithotripsy while maintaining the IRP in a safe range. The choice of surgical position also plays an important role. Compared to the traditional lithotomy position, the head-high lithotomy position has been shown to reduce IRP and complications in prior studies (22), the high plantar lithotomy position facilitates the removal of debris due to gravity. A new modified patient position called the “T-Tilt” can significantly increase SFR, but due to the head-low and foot-high angle during surgery, it may lead to increased IRP, and if the surgery is prolonged, it may increase the incidence of postoperative complications. In addition, according to the fluid dynamics effect theory and the vacuum cleaner effect in continuous flow, stone fragments within 10 mm in front of the endoscope tip, near the sheath mouth, or at the sheath mouth are most effectively attracted and cleared by the gap between the endoscope and the sheath mouth (52), and if FURS and FANS-UAS can operate at the same level, the fragments will deposit at the front end of the FANS-UAS, achieving efficient clearance of fragments (46), in our study, by controlling the distance between the two during surgery, we achieved an immediate stone clearance rate of 90.48%.

In our study, only two patients developed fever after surgery, but both were controlled within 24 h. Apart from this, there was no occurrence of mucosal injury and intraoperative bleeding in our study, which may be related to the soft and bendable cephalic end and the head-high surgical position. Unfortunately, however, two patients developed ureteral lacerations, which led to limitations in stone removal and subsequent treatment. We used the NRS for pain scoring, which has been previously reported for postoperative pain assessment after PCNL or RIRS under secondary local anesthesia after PCNL (53, 54). In our study, most of the patients fell into the mild pain category and did not require medication, and only a few (5.2%) fell into the moderate pain category without severe pain. We used the NRS score for more detailed scoring as compared to the Clavien typology in order to initially assess the pain caused to the patients after surgery.

Our current study also has several limitations. The study utilized a retrospective observational design. As this was our initial clinical experience, the sample size remains small and cannot eliminate potential patient selection bias. Second, due to the higher cost of using fiber optics for pressure measurement compared to traditional pressure measurement and existing pressure measurement technologies, not all center patients received intrarenal pelvic pressure monitoring during surgery, and we are still unable to perform full IRP monitoring, although our fiber-optic manometry is more sensitive and precise in its measurements, we still recommend a more rigorously designed prospective randomized controlled large-case study and existing pressure-measurable UAS for full monitoring.

## Conclusion

Our current study suggests that RIRS with tip-bendable UAS and RTLP is promising in the treatment of upper urinary tract stones. It is both safe and effective, with IRP maintained in a safe range during the procedure. It is recommended that it be well implemented and promoted in primary hospitals as well.

## Data Availability

The original contributions presented in the study are included in the article/Supplementary material, further inquiries can be directed to the corresponding authors.
